# Delayed awakening with apnea as a sign of cerebellar hemorrhage after vestibular schwannoma surgery: A case report

**DOI:** 10.1002/ccr3.3622

**Published:** 2020-12-05

**Authors:** Yosuke Nakadate, Eri Fukasawa, Kodai Ikemoto, Takashi Matsukawa

**Affiliations:** ^1^ Department of Anesthesiology University of Yamanashi Yamanashi Japan

**Keywords:** apnea, cerebellar hemorrhage, delayed awakening

## Abstract

Cerebellar hemorrhage after surgery is a rare but critical complication requiring prompt diagnosis and intervention. However, anesthetics can mask most brain compression signs. Prolonged coma with apnea after tumor resection under general anesthesia may indicate the need for prompt imaging to detect or exclude cerebellar lesions.

## Graphical abstract

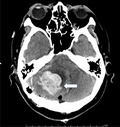

## 1 INTRODUCTION

Cerebellar hemorrhage is a rare but critical complication of surgery, as brain stem compression caused by the hemorrhage can impair vital center functions.^1^ Delayed awakening is one of the complications after surgery involving general anesthesia, and prompt diagnostic imaging studies and surgical intervention are needed in cases with indications of intracranial surgical complications.^2^ However, anesthesia often makes it difficult to detect symptoms arising from intracranial lesions, particularly in the cerebellum. We describe a case of delayed awakening after subtentorial surgery, focusing on the key symptoms and timing of imaging examinations necessary for prompt diagnosis of cerebellar hemorrhage.

## 2 CASE

**FIGURE 1:**
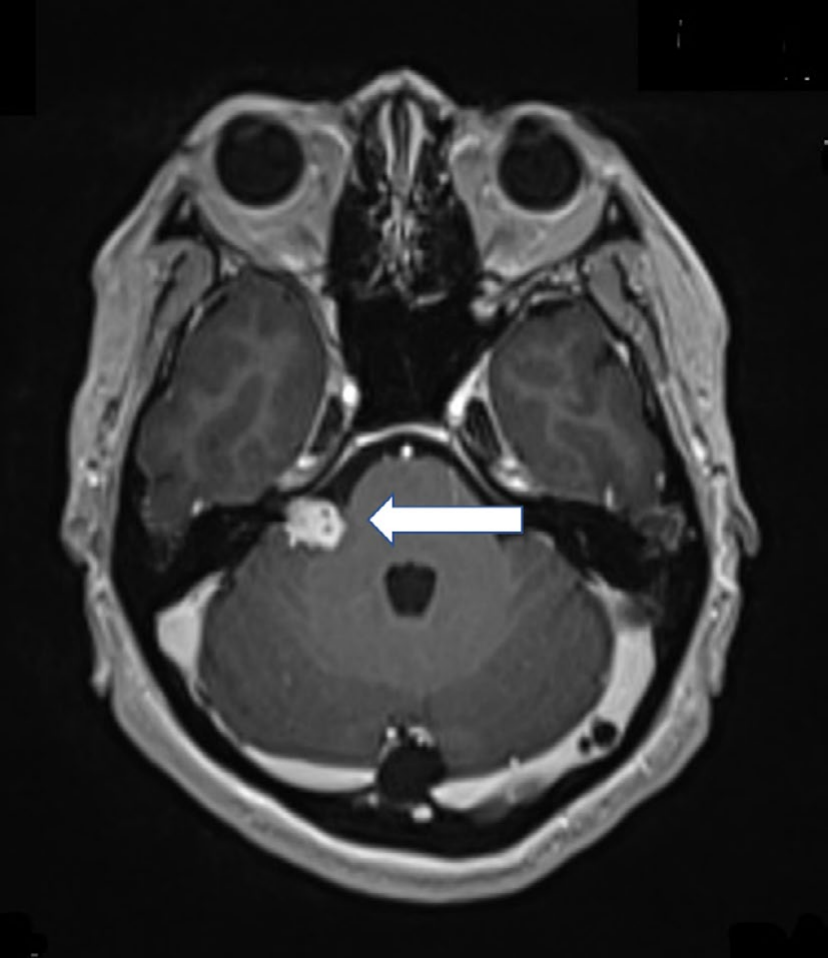
Preoperative brain magnetic resonance imaging. Arrow shows a vestibular schwannoma (16 × 13 mm) at the cerebellopontine angle

A 46‐year‐old woman (height: 162 cm, weight: 64 kg) underwent surgery for the treatment of a 16 × 13 mm, Koos stage 2 vestibular schwannoma at the cerebellopontine angle (Figure 1). She had a current history of smoking but no other medical history and no tumor‐related symptoms. Preoperative blood tests and electrocardiogram findings were normal. Her American Society of Anaesthesiologists Physical Status classification (ASA‐PS) was class 2.

The patient received no premedication. Anesthesia was induced with a target‐controlled infusion (TCI) of propofol (effect compartment concentration 3.0 µg/mL) and remifentanil 0.5 µg/kg/min. Tracheal intubation was performed with the aid of rocuronium 50 mg, and a radial artery line was placed. She was placed in the prone position in pinions and head‐holders.

Anesthesia was maintained uneventfully with TCI of propofol (effect compartment concentration 2.0‐2.5 µg/mL) in oxygen (F_I_O_2_ 0.4) and remifentanil (0.2‐0.3 µg/kg/min). Fentanyl (300 µg) and rocuronium (20 mg) were added during surgery. The surgery was uneventful and the tumor was removed completely; she was placed in the supine position with the pinions removed, and all anesthetics discontinued. The total operation time was 440 minutes. During surgery, her systolic blood pressure (BP) ranged between 100 and 120 mm Hg, heart rate (HR) was between 60 and 70 beats per min (bpm), and SpO_2_ was 99%‐100%.

**FIGURE 2:**
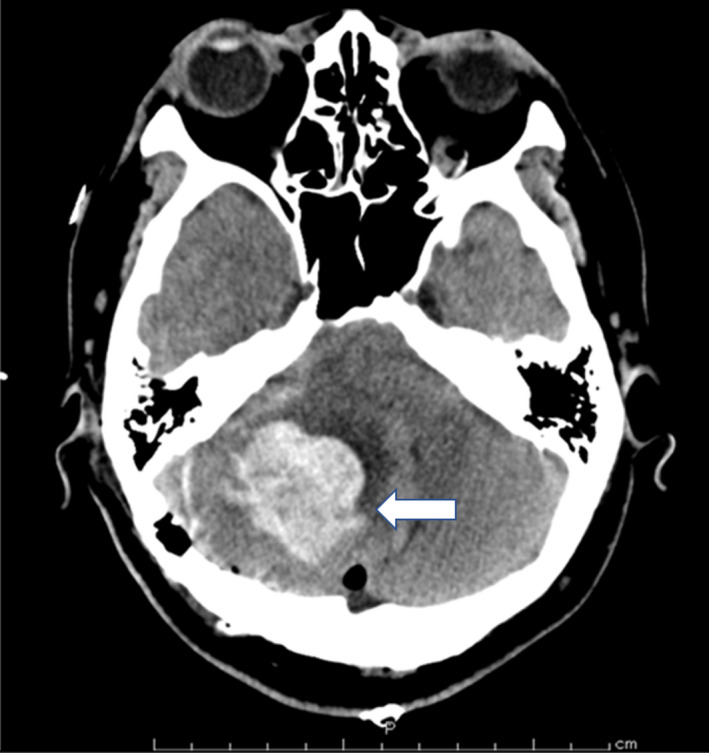
Postoperative brain computed tomography. Arrow shows cerebellar hemorrhage (30 × 37 mm)

For the next 30 minutes, she remained unconscious and unresponsive to painful stimuli and did not cough. Both pupils were 2 mm in diameter. Her temperature was 38.4°C, BP was 145/75 mm Hg, and HR was 90 bpm. Arterial blood gas analysis revealed a pH of 7.415, PaCO_2_ of 44.3 mm Hg, PaO_2_ of 139.2 mm Hg, serum sodium of 137 mmol/L, serum potassium of 3.5 mmol/L, glucose of 164 mg/dL, and ionized calcium of 1.06 mmol/L. Doxapram (40 mg) and sugammadex (200 mg) were administered intravenously, since we assumed that the prolonged anesthetics were possibly the cause for delayed emergence. However, the patient's consciousness did not improve and she was transferred for CT scanning 60 minutes after surgery completion. CT scans revealed a cerebellar hemorrhage (30 × 37 mm) (Figure 2). Immediately after the diagnosis, she was transferred back to the operating room to remove the hematoma. Anesthesia was maintained with a TCI level of propofol (effect compartment concentration 1.0) in oxygen (F_I_O_2_ 0.4) and remifentanil (0.2‐0.3 µg/kg/min). Fentanyl (300 µg) and rocuronium (20 mg) were added. The cause of the hematoma was not detected, and the total operation time for removal was 143 minutes. Fifteen minutes after termination of surgery and discontinuation of anesthetics, the tracheal tube was removed because spontaneous breathing and effective laryngeal reflex had resumed although the patient was still unconscious (Glasgow Coma Scale score [GCS] of E1 VT M4). Her total time under anesthesia for both surgeries was 804 minutes and total blood loss, urine output, and volume of crystalloid were 530 g, 2325 mL, and 3200 mL, respectively.

The patient gradually recovered consciousness following ventricle drainage for hydrocephalus the next day. She was discharged 58 days after the original surgery.

Written informed patient consent was obtained for the publication of this manuscript and accompanying figures. This manuscript adheres to the appropriate EQUATOR guidelines.

## 3 DISCUSSION

This case presented the difficulty in differential diagnosis of delayed awakening and timing of imaging study for the diagnosis in the operating theater.

Delayed awakening after surgery is a serious complication and typically attributed to four factors: patient factors, drug factors, metabolic disorders, and surgical stress and complications.^2^ The incidence of delayed awakening after surgery induced by patient, drug, and metabolic factors has decreased with the development of newer anesthetic techniques, drugs, and monitors. Newer monitors such as the bispectral index monitor (BIS),^3^ neuromuscular monitors^4^ and anesthetics such as desflurane,^3^ rocuronium, and sugammadex^4^ have led to shorter recovery times. These reports^3^, ^4^ suggest that maintaining optimal anesthetic depth can decrease the incidence of delayed awakening induced by drug and patient factors. However, even when monitored appropriately delayed awakening related to patient factors such as genetics, aging, and hepatic disorders may be unavoidable. In these cases, observation until recovery from anesthesia is the principal treatment; some patients may also require anesthetic antagonists.

Newer point‐of‐care testing with arterial gas analyzers and glucometers^5^ allow prompt treatment of metabolic disorders. Accurate and prompt measurements of electrolytes and other parameters can also reduce the occurrence of delayed awakening caused by metabolic factors, including hypo/hyperglycemia and hypo/hypernatremia.

In our patient, delayed awakening was caused by surgical complications. The proportion of intracranial hemorrhage during vestibular schwannoma surgery is 1%‐2% owing to improvements in surgical techniques, with a mortality rate of 0%‐1%.^6^ However, prompt diagnosis and intervention are essential to prevent complications of hemorrhage. Whether and when a patient is transferred for further imaging plays a key role in detecting and preventing life‐threatening complications.

In spontaneous cerebellar hemorrhage patients, radiological signs of brain stem compression, hydrocephalus, a large hematoma size, the presence of clinical signs of brain stem involvement such as lower GCS score, pyramidal tract signs, and others are predictors for poor outcomes.^1^ While postoperative radiological examination is usually conducted after extubation, general anesthesia can mask some signs of intracranial lesions such as paresis, aphagia, pupillary sign, and lower GCS score. Miosis is one of signs for brain stem lesions but it also can be observed under general anesthesia although pupillary signs such as anisocoria in brain hernias and conjugate eye deviation in hemorrhage of the putamen or thalamus can be are key signs for detecting hemorrhage.^7^

Therefore, we should be cautious for impairment signs of vital center functions including respiratory and hemodynamic functions and consciousness as vital centers are located in the brain stem, which is close to the surgical site.

An emergent clinical sign not influenced by anesthetics would be a massive Cushing's sign (bradycardia and hypertension) which suggests increased intracranial pressure.^7^ Although this is an obvious sign for transferring patients to imaging study room, the absence of this sign made us difficult to detect the lesions.

Another sign is prolonged apnea. As respiratory function can be masked by anesthetics, it is important to consider a normal range of awakening time and respiratory recovery time from anesthesia. The standard duration of delayed awakening after surgery has not been defined. House et al defined prolonged emergence as a duration >15 minutes, based on an average time of 5 to 10 minutes between the end of surgery with anesthetics (including propofol, sevoflurane, and desflurane^3^) and extubation.^8^ The average time between discontinuation of anesthesia and extubation was at most 8 to 9 minutes (standard deviation 1‐4 minutes), even in patients undergoing cerebral surgeries where recovery time tends to be slower^9^; therefore, more than 99% of patients were extubated within 21 minutes. Taking into consideration the findings of these studies and earlier reviews reporting that most instances of intraoperative hemorrhage occurred during intracranial or spinal surgery, we should not have observed our patient for more than 20‐30 minutes, apart from local anesthetics injection to the brain stem.^10^

In summary, we describe the case of a patient who showed delayed awakening with prolonged apnea after intracranial surgery. We suggest strongly that prompt imaging to detect or exclude life‐threatening complications should be performed when the patient is in a prolonged coma with apnea after subtentorial tumor resection. It is important to recognize that prolonged apnea, as well as abnormal eye signs after supratentorial surgery or Cushing's sign, may demand prompt diagnosis with appropriate imaging studies after subtentorial tumor surgery.

## CONFLICT OF INTEREST

None.

## AUTHOR CONTRIBUTIONS

YN and EF: involved in initial drafting of manuscript and conception and design of report. KI and TM: contributed to revision of manuscript and critical review of literature. All authors: read and approved manuscript.

## ETHICAL APPROVAL

For this type of study, ethics approval by an institutional review board is not required. Written informed patient consent was obtained for the publication of this manuscript and accompanying figures. This manuscript adheres to the appropriate EQUATOR guidelines.

## Data Availability

The data that support the findings of this study are available from the corresponding author, YN, upon reasonable request.
